# Iatrogenic Botulism After Cosmetic Use of Botulinum Toxin‐A: A Case Series

**DOI:** 10.1002/ccr3.70082

**Published:** 2025-01-06

**Authors:** Mohammad Asadi, Shahin Shadnia, Babak Mostafazadeh, Peyman Erfan Talab Evini, Faraz Zandieh, Mitra Rahimi

**Affiliations:** ^1^ School of Medicine Shahid Beheshti University of Medical Sciences Tehran Iran; ^2^ Toxicological Research Center, Excellence Center & Department of Clinical Toxicology, School of Medicine Shahid Beheshti University of Medical Sciences Tehran Iran

**Keywords:** aesthetic, botulinum toxin, botulism, toxicity

## Abstract

Botulism symptoms after cosmetic botulinum toxin‐A (BTX‐A) injections happen very rarely, and it needs careful attention since it can be life‐threatening. Hence, it is advised to meticulously check the technique, dose, and authenticity of the BTX‐A before injections to reduce the adverse effects.

## 1 Introduction

BTX‐A was not discovered until the early 1900s. BTX‐A is a neurotoxin produced by the *Clostridium botulinum* and is one of the most poisonous biological substances known to man. In 2002, the American Food and Drug Administration (FDA) approved the use of BTX‐A for the cosmetic purpose of temporarily reducing glabellar frown lines [1, 2, 3]. Injections of BTX‐A are now the world's most widely used noninvasive cosmetic procedure. Researchers are becoming more and more interested in discovering the growing number of BTX‐A aesthetic indications [4].

Numerous studies have shown cosmetic BTX‐A injections in the forehead and glabellar areas to be useful, safe, and well‐tolerated [5, 6, 7], as well as indicating the safety and usefulness of axillary injections for hyperhidrosis [5, 8, 9], while most of the adverse effects are temporary and minor [8]. After BTX‐A injection, the toxin spreads in the injection site, but systemic absorption may rarely occur, thus giving rise to botulism symptoms ranging from mild to moderate to severe [10]. Usual presentation symptoms of iatrogenic botulism are generalized muscle weakness, ptosis, dysphagia, diplopia, dysphonia, and rarely, respiratory distress and respiratory failure [11, 12, 13].

Here we report on three patients who presented to Loghman Hakim Hospital, the main referral tertiary center for toxicology cases in Iran. The patients presented with botulism‐like symptoms after aesthetic BTX‐A injections. Interestingly, all the cases happened from November until January 2023. All our cases had BTX‐A injections from MASPORT500 and DYSTON500 brands that are approved by the Iranian FDA, which have never been reported to cause iatrogenic botulism. Unfortunately, as informed by the Iranian FDA, some counterfeit botulinum toxins have been distributed in Iran's market [14], so it is probable that counterfeit BTX‐A with higher potency was used.

## 2 Case History

### 2.1 Case History and Examination

The patient was a 41‐year‐old woman with no history of diseases, medications, alcohol consumption, or smoking, and no specific family history. She presented with dysphagia and difficulty swallowing solids, stridor, vertigo, xerostomia, and proximal muscle weakness. She has had received axillary injections of BTX‐A for hyperhidrosis about 3 weeks prior to the visit; her symptoms started the day after the BTX‐A injection and aggravated in the past 3 weeks.

### 2.2 Case History and Examination

The patient was a 26‐year‐old woman with no history of diseases, medications, alcohol consumption, or smoking, and no specific family history. She presented with proximal muscle weakness in the upper extremities, diplopia, ptosis, vertigo, and difficulty swallowing solids. She had frontalis and orbicularis oculi muscle injections of BTX‐A for aesthetic purposes about 1 week prior to the visit; her symptoms started the day after the BTX‐A injection and aggravated in the past week.

### 2.3 Case History and Examination

The patient was a 38‐year‐old woman with no history of diseases, medications, alcohol consumption, or smoking, and no specific family history. She presented with ptosis and diplopia. She had received injections of BTX‐A to the frontalis and orbicularis oculi muscles for aesthetic purposes about 2 weeks prior to the visit; her symptoms started 5 days after the BTX‐A injection and had aggravated in the past week.

### 2.4 Differential Diagnosis, Investigations, and Treatment

All the cases were admitted immediately and got cardiac monitoring and pulse oximetry, and the botulinum antitoxin regimen was started for them since botulism was the primary differential diagnosis, and other differential diagnoses being myasthenia gravis, radiculoneuropathy, or other diseases of the central nervous system. Routine lab examinations including complete blood count, renal and liver function, venous blood gases, and erythrocyte sedimentation rate were run for all of the cases and indicated no remarkable abnormality. In all three cases, electromyography and nerve conduction studies (Emg‐ncs) for both upper and lower extremities indicated presynaptic nerve block suggesting botulism.

All the cases were treated with botulinum antitoxin with pharmaceutical preparations of ZABOTRIX‐ABE (Masoondarou 10 mL single‐use vials as a Trivalent Type A + B + E A: 50,000, B: 5000, E: 10,000 IU/Vial). The treatment regimen we used in all the cases is as follows: first, an initial dose of 0.5 cc/kg of botulinum antitoxin is given in divided injections (half of the dose given as intravenous infusion in 100 cc normal saline over 30 min and the other half as intramuscular in three different injection sites), then 12–24 h later two‐thirds of the initial dose is given in divided injections (half of the dose given as intravenous infusion in 100 cc normal saline over 30 min and the other half as intramuscular in two different injection sites); finally, 12–24 h after the second dose, one‐third of the initial dose is given as divided injections (half of the dose given as intravenous infusion in 100 cc normal saline over 30 min and the other half as intramuscular). Case 3 was further treated with oral administration of pyridostigmine 60 mg tablet every 6 h for 10 days since she still had mild ptosis of the eyelids.

### 2.5 Outcome

After treatment with the mentioned botulinum antitoxin regimen, we performed a thorough physical examination and review of systems of the patients. Case 1 showed regain of muscle strength, resolving of swallowing problems, and improvement of the xerostomia. Case 2 showed regain of muscle strength, improvement of the vertigo, resolving of the diplopia, and resolution of swallowing problems. Case 3 indicated alleviation of ptosis and diplopia. The patients were discharged in good general condition.

## 3 Discussion

Numerous studies show the usefulness, good tolerability, and safety of BTX‐A for treating hyperhidrosis [6, 7, 15] and forehead wrinkles [5, 8, 9]. The usual adverse effects after BTX‐A injection are hematoma, pain, allergic reactions, flu‐like symptoms, and rarely botulism [16, 17].

There have been case reports globally of iatrogenic botulism after injections of BTX‐A [11, 12, 13, 18, 19, 20, 21, 22]. Richardson et al. reported a case of iatrogenic botulism after forehead and axillary BTX‐A injections, which was resolved by botulinum antitoxin treatment [23]. Ghasemi et al. reported two cases of iatrogenic botulism after axillary injections for hyperhidrosis in therapeutic doses [18]. In 2018, Bai L et al. presented an analysis of 86 iatrogenic botulism cases caused by the cosmetic injection of BTX‐A and showed the effectiveness of antibotulinum toxin treatment [23]. Fan et al. reported the usefulness and safety of delayed botulinum antitoxin treatment after cosmetic BTX‐A injections [11].

Iatrogenic botulism is usually presented with generalized weakness, dysphagia, ptosis, diplopia, dysphonia, and rarely respiratory distress and the symptoms usually occur 0–36 days after BTX‐A injections [12, 13, 20, 21].

The most common symptoms that our cases presented with were proximal muscle weakness in the upper extremities, difficulty swallowing solids, and ptosis. None of the patients experienced respiratory distress or a drop in blood oxygen saturation. Two of our cases had cosmetic injections for forehead and periorbital wrinkles, and one of them had axillary injections for hyperhidrosis. In all cases, there were no remarkable lab result abnormalities, and the symptoms of botulism were alleviated after the mentioned regimen of botulinum antitoxin treatment.

The cases and adverse effects were reported to the Iranian FDA for further investigation.

## 4 Conclusion

Our three cases highlighted manifestations of iatrogenic botulism after BTX‐A injections. Since the injections were common cosmetic forehead, periorbital, and axillary injections, clinicians are advised to carefully check the dose and authenticity of their BTX‐A.

## Author Contributions

**Mohammad Asadi:** conceptualization, data curation, writing – original draft, writing – review and editing. **Shahin Shadnia:** supervision, writing – review and editing. **Babak Mostafazadeh:** supervision, writing – review and editing. **Peyman Erfan Talab Evini:** supervision, writing – review and editing. **Faraz Zandieh:** data curation. **Mitra Rahimi:** conceptualization, supervision, writing – review and editing.

## Consent

Written informed consent was obtained from the patients to publish this report, in accordance with the journal's patient consent policy. A copy of the written consent is available for review by the editor‐in‐chief of this journal on request.

## Conflicts of Interest

The authors declare no conflicts of interest.

## Data Availability

The medical records of all the patients are collected and available.
